# Drug seller adherence to clinical protocols with integrated management of malaria, pneumonia and diarrhoea at drug shops in Uganda

**DOI:** 10.1186/s12936-015-0798-9

**Published:** 2015-07-16

**Authors:** Phyllis Awor, Henry Wamani, Thorkild Tylleskar, Stefan Peterson

**Affiliations:** Department of Global Public Health and Primary Health Care, Centre for International Health, University of Bergen, PO Box 7800, 5020 Bergen, Norway; Department of Community Health and Behavioural Sciences, School of Public Health, College of Health Sciences, Makerere University, PO Box 7072, Kampala, Uganda; Global Health, Karolinska Institutet, Stockholm, Sweden; International Maternal and Child Health Unit, Uppsala University, Uppsala, Sweden

## Abstract

**Background:**

Drug shops are usually the first source of care for febrile children in Uganda although the quality of care they provide is known to be poor. Within a larger quasi-experimental study introducing the WHO/UNICEF recommended integrated community case management (iCCM) of malaria, pneumonia and diarrhoea intervention for community health workers in registered drug shops, the level of adherence to clinical protocols by drug sellers was determined.

**Methods:**

All drug shops (N = 44) in the intervention area were included and all child visits (N = 7,667) from October 2011–June 2012 to the participating drug shops were analysed. Drug shops maintained a standard iCCM register where they recorded the children seen, their symptoms, diagnostic test performed, treatments given and actions taken. The proportion of children correctly assessed and treated was determined from the registers.

**Results:**

Malaria management: 6,140 of 7,667 (80.1%) total visits to drug shops were of children with fever. 5986 (97.5%) children with fever received a malaria rapid diagnostic test (RDT) and the RDT positivity rate was 78% (95% CI 77–79). 4,961/5,307 (93.4%) children with a positive RDT received artemisinin combination therapy. Pneumonia management: after respiratory rate assessment of children with cough and fast/difficult breathing, 3,437 (44.8%) were categorized as “pneumonia”, 3,126 (91.0%) of whom received the recommended drug—amoxicillin. Diarrhoea management: 2,335 (30.5%) child visits were for diarrhoea with 2,068 (88.6%) correctly treated with oral rehydration salts and zinc sulphate. Dual/Triple classification: 2,387 (31.1%) children had both malaria and pneumonia and 664 (8.7%) were classified as having three illnesses. Over 90% of the children with dual or triple classification were treated appropriately. Meanwhile, 381 children were categorized as severely sick (with a danger sign) with 309 (81.1%) of them referred for appropriate management.

**Conclusion:**

With the introduction of the iCCM intervention at drug shops in Eastern Uganda, it was possible to achieve high adherence to the treatment protocols, which is likely compatible with increased quality of care.

**Electronic supplementary material:**

The online version of this article (doi:10.1186/s12936-015-0798-9) contains supplementary material, which is available to authorized users.

## Background

Every year, over 30 million cases of malaria, pneumonia and diarrhoea in children go untreated in Uganda [1]. Under-five mortality from these three illnesses is 13, 15 and 9%, respectively [2]. This large treatment gap and high under-five mortality can be reduced by ensuring high access to life saving preventive and curative services for these illnesses. However, access to health services in Uganda is variable, ranging from 7 to 100% in different parts of the country. Even where health facilities are available, low staffing, lack of necessary supplies and equipment and various health system challenges make access to basic care uncertain [1]. As a result of gaps within the public health sector, the private sector—dominated by drug shops in rural areas—has proliferated and is a major source of care for sick children [3]. More than half of sick children in Uganda seek care at the level of a drug shop when ill [4, 5]. However, without any interventions at this level, the quality of care has been documented to be poor [6, 7].

Meanwhile, evidence shows that health workers, both public and private generally have low adherence to clinical protocols. For example, health workers in malaria endemic areas continue to prescribe artemisinin combination therapy to patients whose malaria diagnostic test result is negative, for various reasons including mistrust of test results [8–11]. Lower level health workers for example community health workers have been shown to follow clinical protocols more strictly [12, 13]. However, the adherence of drug shop attendants to treatment protocols for malaria, pneumonia and diarrhoea is largely unknown. Given the role of drug shops in the care for children in Uganda and other low-income countries, it is necessary to understand adherence to treatment protocols in this group of health providers.

The objective of this study was to determine the level of adherence by drug shop attendants in eastern Uganda, to the integrated community case management (iCCM) of malaria, pneumonia and diarrhoea treatment guidelines.

## Methods

This analysis was done with data collected during a quasi-experimental study determining the feasibility and effect on access and appropriateness of treatment with the introduction of the integrated community case management of malaria, pneumonia and diarrhoea intervention at drug shops. The study site, intervention, methods and results of the main study are published elsewhere [4]. For this paper, all drug shops (N = 44) in the intervention area were included and all child visits (N = 7,667) from October 2011–June 2012 to the participating drug shops were analysed.

### Data collection

Drug shops maintained a standard iCCM register (see Additional file 1) where they recorded the children seen, their symptoms (fever, cough, fast/difficult breathing etc.), diagnostic test performed, the test results, treatment given and actions taken. For every child with fever, drug shop attendants were trained to perform a malaria rapid diagnostic test (RDT) and record the test result as positive or negative. Similarly for every child with cough and fast/difficult breathing, the drug shop attendants were trained to count the respiratory rate using the UNICEF respiratory rate (RR) counter and record the findings. Based on the standard iCCM sick-child-job-aid provided (see Additional files 2, 3), this child was then categorized as having pneumonia or not, taking into consideration the age and respiratory rate. Children with diarrhoea were also recorded in the register. After assessment, appropriate treatment was then given to the children.

### Quality control of diagnostic testing and treatment

Quality control was done during the main study through support supervision. Both the local district drug inspector and a study nurse supervised the drug shops separately. After the iCCM training, a full time study nurse was employed to provide support supervision to the drug shop attendants. Initial support supervision was weekly at each drug shop and this was later reduced to monthly as less support became necessary. During the supervision visits, the study nurse observed the drug shop attendants manage children from the initial diagnosis of malaria, pneumonia or diarrhoea with utilization of the respective diagnostic test (RDT or RR counter), to the provision of appropriate treatment. The study nurse then discussed with the drug shop attendant the positive aspects and weaknesses observed during the management of the child. Continuous training was also provided to the drug shop attendant as necessary. More details of the support supervision process and results from some of the direct observation exercises have been previously published [4].

### Sample size

To determine prevalence of adherence to clinical protocol from the registers, we used all records of children seen at the drug shops during the entire study period (8 months). The total sample size was 7,667 child visits to the drug shops.

### Analysis

Data was double-entered in epi data [14] and was analysed using STATA, version 12 [15]. During the analysis, the symptoms and test results recorded in the iCCM registers were utilized, and every child was re-classified according to the iCCM algorithm. For children recorded as having fever, the analysis checked that an RDT result was recorded and whether appropriate treatment was given. For children recorded as having cough and fast/difficult breathing, the analysis checked that a respiratory rate was recorded, then this respiratory rate was reclassified as fast or not (thus presence or absence of ‘pneumonia’), based on the age of the child, as required by the guidelines. The appropriateness of treatment provided for pneumonia was assessed. For all children recorded as having diarrhoea, the analysis checked whether the recorded treatment was appropriate. Finally, the quality of integrated management of several concurrent symptoms was assessed. The proportion of children receiving a single, double, or triple illness classification was determined, and then the proportion of these children correctly assessed and treated was calculated. Confidence intervals around the estimates are provided.

### Ethics, consent and approvals

Ethical approval was obtained from Uganda National Council of Science and Technology (# HS 1184), the Uganda National Drug Authority (# 0456/ID/NDA) and Makerere University School of Public Health (# 166). Written consent was obtained from the drug sellers who participated in this study.

## Results

A total of 7,667 children were seen at the drug shops during the eight months study period, from October 2011–May 2012. Of all recruited drug shops (N = 44), two dropped out of the study in the initial months and another two were closed permanently, leaving 40 participating drug shops for the duration of the study. While half (22/40) of the drug shops saw almost 90% of the children, just five drug shops saw nearly half of all the children. Table 1 shows the total number of children seen every month. Over the first 3 months the percentage of children seen at the drug shops was constant at about 11% of the total number of children seen. Over the remaining months of the study, there was an increase in the number of children seen (p ≤ 0.001).

**Table 1:** Total number of children seen at drug shops by month of study

Month	Frequency	Percent	Test for trend
October 2011	858	11.2	P ≤ 0.001
November	900	11.7	
December	883	11.5	
January 2012	784	10.2	
February	950	12.4	
March	1,291	16.9	
April	850	11.1	
May	1,151	15.0	
Total	7,667	100	

The children seen at the drug shops had a mean age of 21 months (range 0–84) and 47% were 12 months and below, (Table 2). Eighty percent (6,140/7,667) of all the children were recorded as having fever. Of these, 5986 (98%) were tested for malaria with an RDT and the overall RDT positivity rate was 78% (95% CI 77–79). Further, 94% of all children with a positive RDT test received the recommended malaria treatment, ACT.

**Table 2:** Classification and treatment of children attending drug shops

Characteristic	Proportion	95% Confidence Intervals
Child age		
Up to 1 year	3,596 (46.9%)	
Greater than 1 year	4,071 (53.1%)	
Child sex—male	3,732 (48.8%)	
Diarrhoea		
Proportion with diarrhoea	2,335/7,667 (30.5%)	29.4–31.5
Received ORS packet	2,068/2,335 (88.6%)	87.3–89.9
Fever		
Proportion with fever	6,140/7,667 (80.1%)	79.2–81.0
Fever and received Rapid Diagnostic test (RDT)	5,986/6,140 (97.5%)	
Fever and RDT positive	5,096/6,140 (83.0%)	82.1–83.9
Received an RDT (ALL RDT tests done)	6,801/7,667 (88.7%)	
RDT positive (overall RDT positivity)	5,307/6,801 (78.0%)	77.0–79.0
RDT positive and got ACT	4,961/5,307 (93.5%)	92.8–94.1
Any ACT dispensed	5,218/7,667 (68.1%)	67.0–69.1
Pneumonia		
Respiratory rate counted (all RRs counted)	5,203/7,667 (67.9%)	
Proportion with pneumonia	3,437/7,667 (44.8%)	
Pneumonia and amoxicillin dispensed	3,126/3,437 (91.0%)	
Any amoxicillin dispensed	3364/7,667 (43.9%)	42.8–45.0
Danger signs		
Proportion with any danger sign	381/7,667 (5.0%)	
Danger sign and referred	309/381 (81.1%)	
Outcome		
Referred	477/7,667 (6.2%)	5.7–6.8
Adverse drug reaction	2/7,667	
Died	2/7,667	

In this study, 5,203 (67.9%) were assessed for fast breathing and 45% (3,437/7,667) were classified as having ‘pneumonia’ according to the MoH/WHO ICCM guidelines. The mean respiratory rate recorded was 48 breaths per minute (standard deviation 11.9). Of the children classified as having pneumonia, 91% received the recommended drug, amoxicillin. Thirty-one percent (2,335/7,667) of all the children seen at the shops had diarrhoea and 89% of these were recorded as having received the recommended treatment, ORS/zinc. Further, a total of 5% of children in the sample were classified as severely ill (having a danger sign) and the majority of these children were reported to have been referred to the health centre.

The proportion of children in the sample who were classified with both malaria and pneumonia was 31% and most of these (93%) were recorded as having received both ACT for malaria and amoxicillin for pneumonia—Table 3. Meanwhile, 9% of all the children seen at drug shops were found to have all three illnesses (malaria, pneumonia and diarrhoea) and the majority was treated appropriately.

**Table 3:** Management of double or triple illnesses in children, at drug shops

Both malaria and pneumonia	
Children with both malaria and pneumonia	2,387/7,667 (31.1%)
Received ACT	2,237/2,387 (93.7%)
Received Amoxicillin	2,211/2,387 (92.6%)
Malaria, pneumonia and diarrhoea	
Children with all 3 illnesses	664/7,667 (8.7%)
Received ACT	624/664 (94.0%)
Received Amoxicillin	607/664 (91.4%)
Received ORS/zinc	580/664 (87.3%)

## Discussion

This analysis of treatment registers shows high adherence to the iCCM guidelines by drug shop attendants. Over 90% of all children with pneumonia-related symptoms, fever or diarrhoea were appropriately assessed, classified and treated according to guidelines. This is likely to represent an enormous step up in quality of care as illustrated by our previous paper where exit interviews and household surveys were used to assess appropriateness of treatment of children at the drug shops. That study found 3–13 times better management of children at drug shops with the introduction of iCCM [4]. Beside these two studies, there is limited additional evidence on the utilizing the iCCM strategy at the level of drug shops as studies at drug shops in low income countries have focused on single diseases especially malaria, at the expense of other febrile illness [16].

Similarly high levels of adherence to malaria RDT results by community health workers (97%) have been demonstrated in Uganda and other African countries [12, 13, 17]. However, in two of these studies, the classification and treatment of pneumonia by CHWs was demonstrated to be poor with for example, only 40% of children with pneumonia symptoms being prescribed an antibiotic [12]. One child in three (31%) received treatment for both malaria and pneumonia. While this confirms earlier findings by Källander et al. of a 30% symptom overlap [18], it also means that there was little further reduction in drug use from inclusion of RDTs compared to presumptive management. However, this was a high malaria transmission area with estimated parasite prevalence of 60% in school-age children [19, 20] and the RDT positivity rate at the drug shops from this study was 77%. Limited penetration of malaria bed nets in this high malaria transmission area is likely to explain the high RDT positive rate, which will further have been augmented by the inherent problem of false positivity with malaria RDT—due to persistence of *Plasmodiumfalciparum* histidine-rich protein 2 (*Pf*HRP2) antigens four up to 1 month in the blood stream, following elimination of parasites [21, 22]. In comparison, Nankabirwa et al. reported 51% malaria parasite prevalence using the gold-standard, microscopy, in children 0–59 months at public health facilities in regions with moderate-high malaria transmission in Uganda [23].

The high malaria positivity rate and the 45% of children whose respiratory rate was found high after assessment, meant that the drug shops could have continued, and perhaps even increased, the number of drugs sold per child, which coupled with the 25% increased utilization of these drug shops [4] likely increased their total financial turnover. Exactly how this affected total profits is not clear since we do not know profit margins of drugs earlier sold, although it was noted anecdotally that shopkeepers were almost uniformly happy saying “this has increased my business”. However, how drug shop attendants would react in lower malaria endemic settings where a lower proportion of RDTs were positive and an increasing proportion of children are supposed to be treated with lower-profit-margin paracetamol is not clear, but needs to be subject to further study.

While we followed up 40 drug shops for the entire study period, care seeking tended to concentrate around particular drug shops. Five drug shops saw nearly half of all the children, during the entire study period. While this was unexpected, it is logical that parents may prefer certain characteristics of health workers/drug shop attendants and thus choose to take their children to these health workers. This concentration of care seeking has the potential to allow closer supervision, and also gaining more experience.

The discourse around governance of the private sector in pluralistic health systems emphasizes balancing both regulation and incentives [24, 25]. Regulation alone may be insufficient as state regulation is weak and the processes for self-regulation are lacking. Incentives available to drug sellers for example through this iCCM intervention can improve quality of care, but there are limits to positive incentives alone. Community awareness to enable demand for quality service is also very important. This will likely need to involve aspects of asking for, and respecting test results, and an acceptance of treatment of children not with anti-malarials or antibiotics, but with for example paracetamol only if they have no malaria and no pneumonia.

This study has two important limitations that should be considered. First, the analysis is based on data from drug shop registers, which was not validated. However, this data was collected during a prospective experimental study and these results are very similar to results from the main study which used different data collection techniques like exit interviews and household surveys [4]. Secondly, the case definition of “pneumonia” in this study is not for confirmed pneumonia diagnosis, but for the iCCM classifications of ‘pneumonia’—cough with fast/difficult breathing. This classification tends to result in more children being classified as having “pneumonia” than in reality.

## Conclusion

This paper shows that with the introduction of the iCCM intervention at drug shops in Eastern Uganda, it was possible to achieve high adherence to the treatment protocols, which is likely compatible with increased quality of care.

## Additional files

Additional file 1: The ICCM register: This is a copy of the standard book, where drug shop attendants (and community health workers) record all children seen with symptoms of malaria, pneumonia or diarrhoea. Additional file 2: The sick child job aid 1. This is a visual aid that contains the iCCM treatment algorithm. It is printed in A3 size and displayed in the drug shop for easy reference when managing children. Additional file 3: The sick child job aid 2. This is a visual aid that contains the iCCM treatment algorithm. It is printed in A3 size and displayed in the drug shop for easy reference when managing children.
