# Supplementary material for: Drug seller adherence to clinical protocols with integrated management of malaria, pneumonia and diarrhoea at drug shops in Uganda
Source: Malar J. 2015 Jul 16;14:277. doi: 10.1186/s12936-015-0798-9 (PMC4502601; doi:10.1186/s12936-015-0798-9)
Supplement: Additional file 1: — The ICCM register: This is a copy of the standard book, where drug shop attendants (and community health workers) record all children seen with symptoms of malaria, pneumonia or diarrhoea. [file 12936_2015_798_MOESM1_ESM.pdf]

ICCM A2L DRUGSHOP REGISTER FOR CHILDREN 5 YEARS AND BELOW

District: ______________________ Sub County: ______________________ Facility: ______________________ Reporting Month and Year: ______________________

GENERAL INFO								PROBLEM					TREATMENT								OUTCOME			
													Diarrhoea			Fast Breathing		Fever						
Date	Patient Name	SEX		AGE	Respiratory Rate (breaths/ minute)	RDT Results		Diarrhoea	Fast Breathing	Fever	Danger Sign	Treated within 24 hrs	ORS	ZINC		AMOXICILLIN		ACT		Referred	Recover-ed	Died	Bad Medicine Reaction	Un-known
		M	F			+	–							0 to 6 months	7 mths to 5 yrs	4 to 11 months	1 to 5 years	4 mths to 2 yrs	3 to 7 years					
													packets	1/2 tablet	1 tablet	Red	Green	Yellow	Blue					
							Total					Total												

DIARRHOEA SUMMARY

Add up the total numbers from above and write in each box below.

Diarrhoea	With danger sign	Recovered	Referred	Died

ORS	Packets	ZINC	1/2 tablet (0 to 6 months)	1 tablet (7 months to 5 yrs)
Received:		Received:		
Given out:		Given out:		
Remaining:		Remaining:		

FAST BREATHING SUMMARY

Add up the total numbers from above and write in each box below.

Fast Breathing	With danger sign	Recovered	Referred	Died

AMOXICILLIN	Red (4 to 11 months)	Green (1 to 5 years)
Received:		
Given out:		
Remaining:		

FEVER SUMMARY

Add up the total numbers from above and write in each box below.

Fever	With danger sign	Recovered	Referred	Died

ACT	Yellow (4 months to 2 years)	Blue (3 to 7 years)
Received:		
Given out:		
Remaining:		

RDT SUMMARY

Received:	
Used:	
Remaining:	

Filled by: Name .... Signature .... Title .... Date ....
