# Supplementary material for: Drug seller adherence to clinical protocols with integrated management of malaria, pneumonia and diarrhoea at drug shops in Uganda
Source: Malar J. 2015 Jul 16;14:277. doi: 10.1186/s12936-015-0798-9 (PMC4502601; doi:10.1186/s12936-015-0798-9)
Supplement: Additional file 2: — The sick child job aid 1. This is a visual aid that contains the iCCM treatment algorithm. It is printed in A3 size and displayed in the drug shop for easy reference when managing children. [file 12936_2015_798_MOESM2_ESM.pdf]

1. ASK THE CAREGIVER

How old is the child?

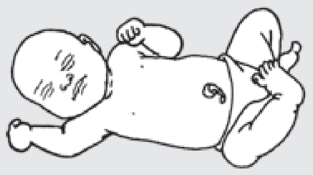
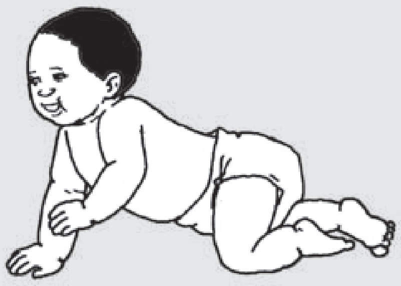
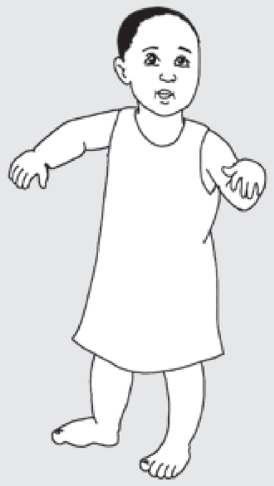
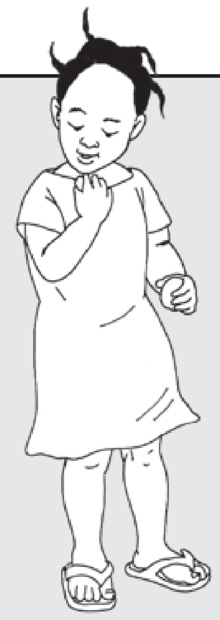

☐ **0 - 7 days** ☐ **2 - 11 months** ☐ **1 - 2 years** ☐ **3 - 5 years**

2. ASK - CHILD'S PROBLEMS

Does the child have cough, diarrhoea or fever?

If yes, for how long?

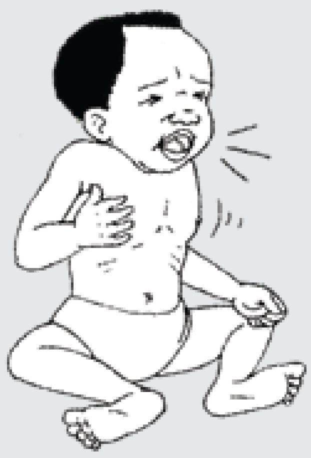
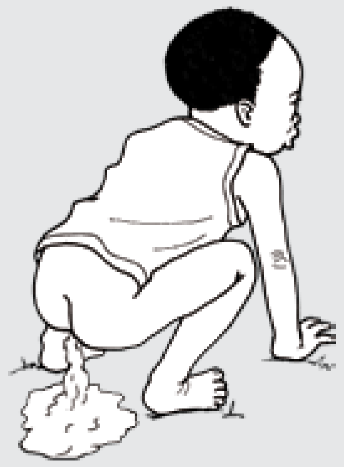
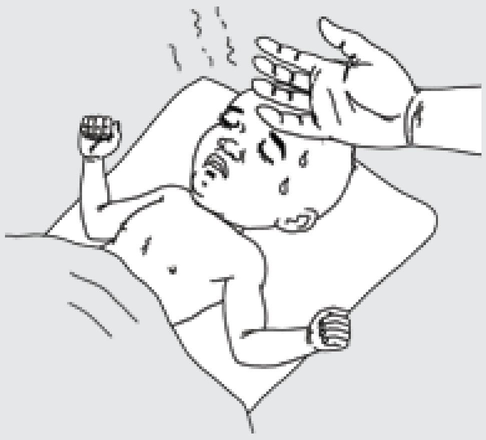

☐ **Cough** ☐ **Diarrhoea** ☐ **Fever**

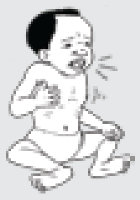

If **Cough** is present, check for **Fast Breathing**.

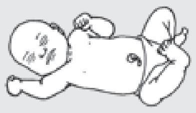

0 – 7 days

60 or more

breaths per minute

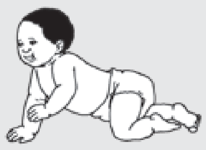

2-11 months

50 or more

breaths per minute

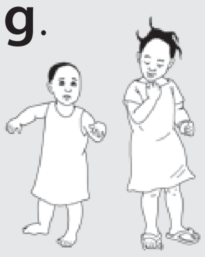

1-5 years

40 or more

breaths per minute

THE COMPLETE SICKCHILD JOBAID

THE REPUBLIC OF UGANDA Ministry of Health

Medicines for Malaria Venture

a2l ACCESS 2 LIFE

pace Interventions for Health Impact

3. ASK AND LOOK FOR DANGER SIGNS AND REFER

Refer to Step 4a because children with some of these danger signs need pre-referral treatment.

Any child or newborn with...

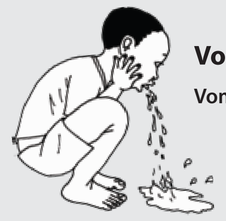

Vomiting ☐
Vomits everything

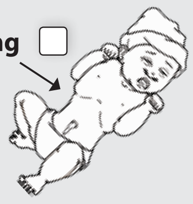

Chest in-drawing ☐

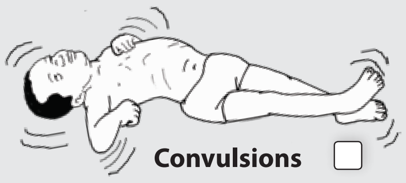

Convulsions ☐

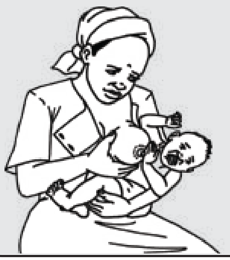

Not able to breastfeed or drink ☐

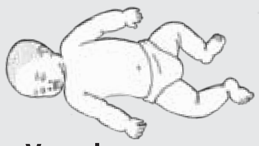

Very sleepy or unconscious ☐

A newborn with...

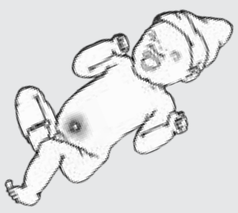

Infected umbilical cord ☐

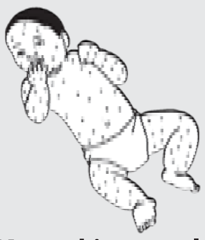

Many skin pustules ☐

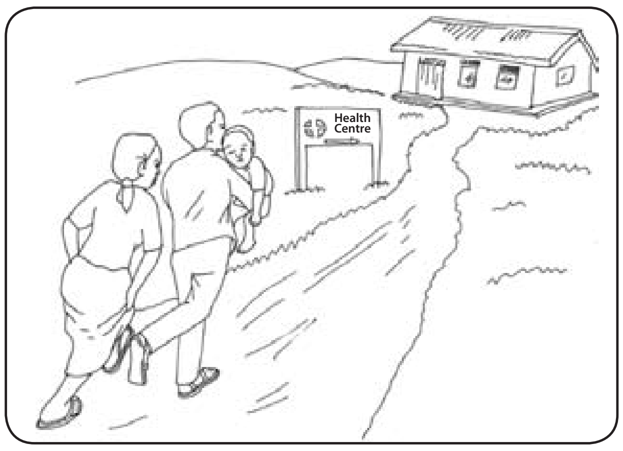

A child with...

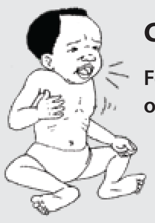

Cough ☐
For 21 days or more

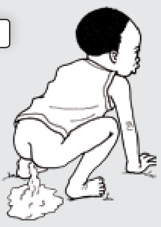

Diarrhoea ☐
For 14 days or more, or with blood

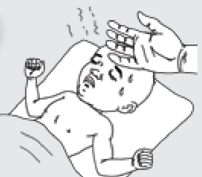

Fever ☐
For 7 days or more

4a. PRE-REFERRAL TREATMENT

If the child has any of the danger signs below, give the following treatment:

Diarrhoea
For 14 days or more, or with blood in the stool

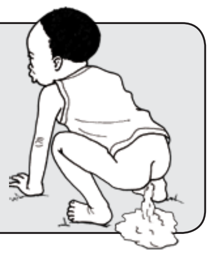

☐ Begin giving the child ORS before you refer. Advise caregiver to continue giving ORS to the child on the way to the health centre.

Chest In-drawing or Fast Breathing
With a Danger Sign

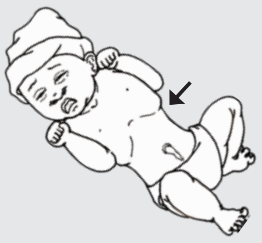

☐ Give first dose of Amoxicillin before you refer.

2 - 11 months: 2 tabs from RED PACK
1 - 5 years: 3 tabs from GREEN PACK

Fever
For 7 days or more

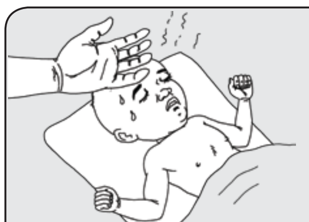

☐ Give first dose oral Anti-Malarial ACT.

4 months - 2 years: 1 tab from YELLOW PACK
3 - 5 years: 2 tabs from BLUE PACK

Fever and a General Danger Sign

☐ Give Rectal Artesunate.

4 - 11 months: 1 cap
1 - 3 years: 2 caps
4 - 5 years: 4 caps

☐ Help caregiver give dose.

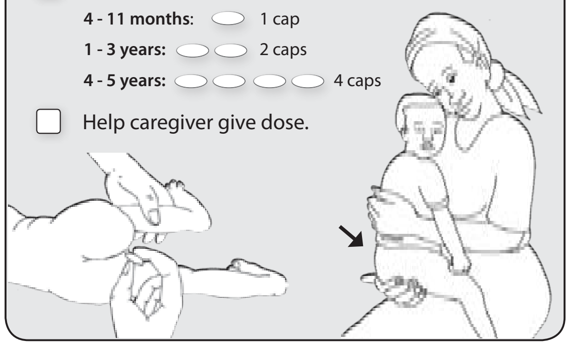

Newborn
With a Danger Sign

☐ Give first dose of Amoxicillin.

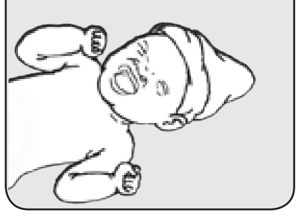

4b. TREAT AND ADVISE

If the child has any of the symptoms below with NO danger signs, give the following treatment:

Cough
With fast breathing for less than 21 days

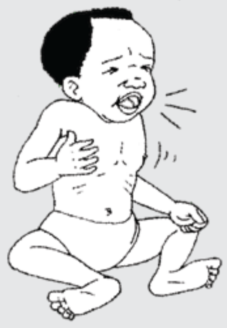

☐ Give oral antibiotic – Amoxicillin.

☐ Advise caregiver to give:

2 - 11 months: 2 tabs twice a day for 5 days from RED PACK
1 - 5 years: 3 tabs twice a day for 5 days from GREEN PACK

☐ Help caregiver give first dose now.

Diarrhoea
For less than 14 days, and without blood in stool

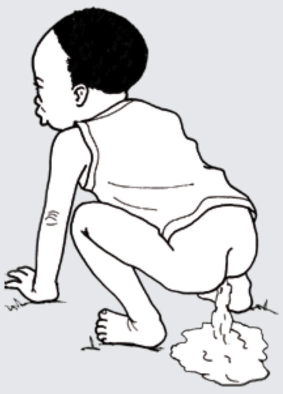

☐ **Give ORS.** Help caregiver give child ORS solution in front of you until the child is no longer thirsty.

☐ **Give caregiver 2 ORS packets to take home.** Advise caregiver to give as much as child wants, but at least ½ cup ORS solution after each loose stool.

☐ **Give zinc supplement.** Give 1 dose daily for 10 days.

2 to 6 months: ½ a tab once a day for 10 days
6 months to 5 years: 1 tab once a day for 10 days

☐ Help caregiver give first dose now.

Fever
Less than 7 days

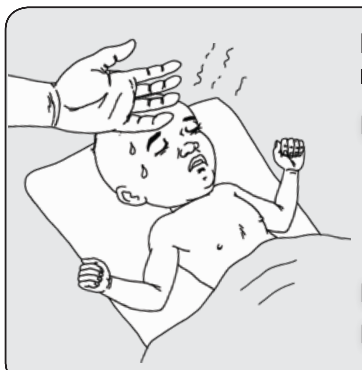

☐ Give oral Anti-Malarial ACT.

4 months to 2 years: 1 tab twice a day for 3 days from YELLOW PACK
3 years to 5 years: 2 tab twice a day for 3 days from BLUE PACK

☐ Help caregiver give first dose now.

☐ Advise caregiver on use of a bed net (LLIN).

THE COMPLETE SICKCHILD JOBAID

THE REPUBLIC OF UGANDA Ministry of Health

Medicines for Malaria Venture

a2l ACCESS 2 LIFE

pace Interventions for Health Impact
