# Supplementary material for: Drug seller adherence to clinical protocols with integrated management of malaria, pneumonia and diarrhoea at drug shops in Uganda
Source: Malar J. 2015 Jul 16;14:277. doi: 10.1186/s12936-015-0798-9 (PMC4502601; doi:10.1186/s12936-015-0798-9)
Supplement: Additional file 3: — The sick child job aid 2. This is a visual aid that contains the iCCM treatment algorithm. It is printed in A3 size and displayed in the drug shop for easy reference when managing children. [file 12936_2015_798_MOESM3_ESM.pdf]

5. ADVICE FOR ALL CHILDREN TREATED AT HOME

Give more fluids and continue feeding

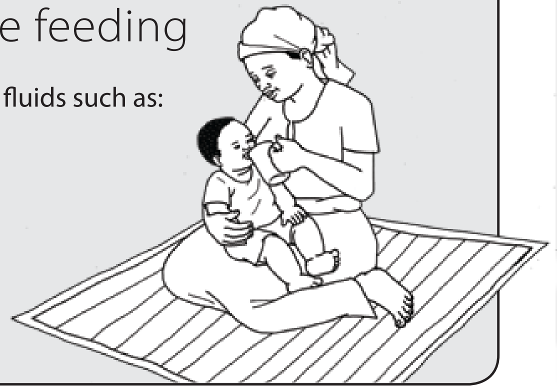

- [ ] Advise caregiver to give plenty of homemade fluids such as:
  - Clean water
  - Soup
  - Yoghurt drinks
  - Light porridge
  - ORS

Go to the health facility if...

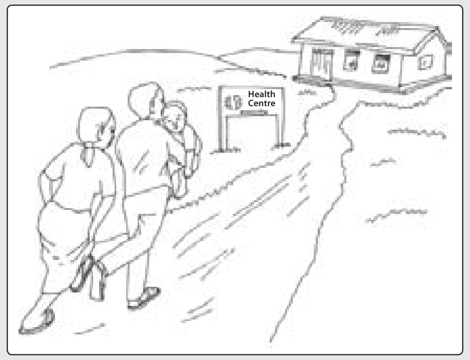

- [ ] Advise to go to the health facility if the child:
  - Cannot drink or feed
  - Has blood in stool
  - Becomes sicker
  - Develops any other danger sign
  - Has not improved in 2 days

Sleep under a net

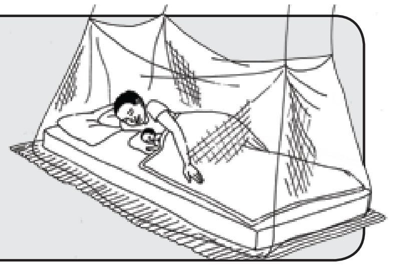

- [ ] Advise caregiver to be sure the child sleeps under a mosquito net.

Follow up child in 3 days

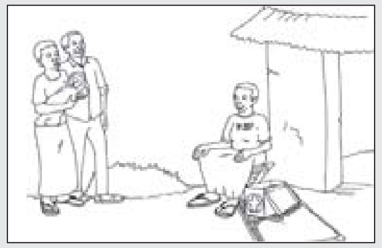

- [ ] Decide if you (the VHT member) will go to the child or if the caregiver will bring the child to you.

6. ROUTINE CARE FOR THE NEWBORN

Keeping the baby warm

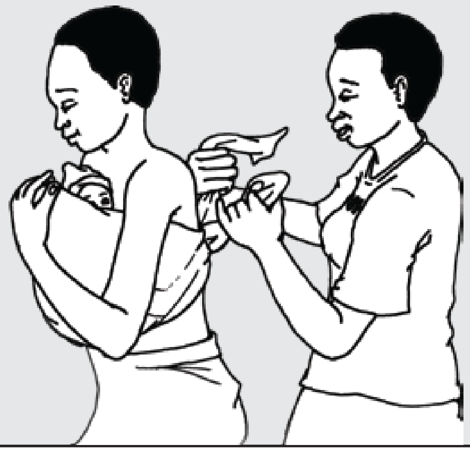

Advise the caregiver on the following:

- [ ] Wrap the baby in warm, dry clothes, including the head and feet.
- [ ] Put the baby in skin-to-skin contact with the mother, as shown in the picture.
- [ ] Delay the baby's first bath until after 24 hours.

Breastfeeding exclusively

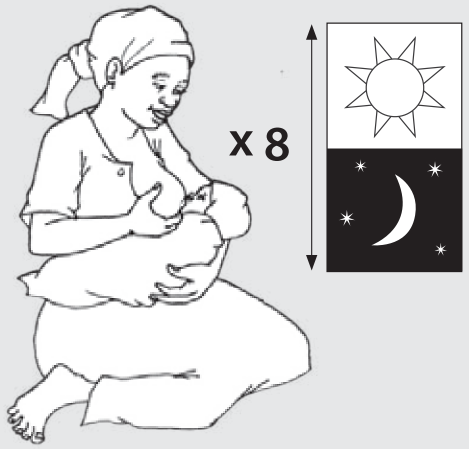

Advise the mother on the following:

- [ ] Start breastfeeding immediately after birth.
- [ ] Feed the baby on only breast milk, on demand, at least 8 times a day.
- [ ] Ensure the baby is well positioned and attached on the mother's breast.

Skin and cord care

Advise the caregiver on the following:

- [ ] Wash hands before handling the baby.

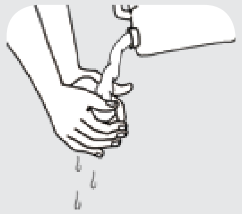

- [ ] Do not apply anything on the cord. Leave the cord dry and open.

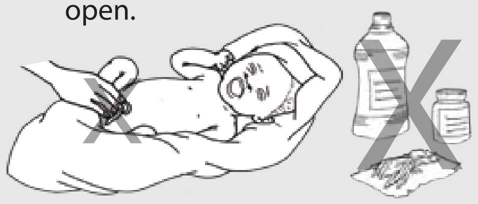

- [ ] Bathe the baby with clean soap and water.
